# Erythropoietin Protects Cardiomyocytes from Cell Death during Hypoxia/Reperfusion Injury through Activation of Survival Signaling Pathways

**DOI:** 10.1371/journal.pone.0107453

**Published:** 2014-09-19

**Authors:** Asiya Parvin A, Raj Pranap A, Shalini U, Ajay Devendran, John E. Baker, Anuradha Dhanasekaran

**Affiliations:** 1 Centre for Biotechnology, Anna University, Chennai, Tamil Nadu, India; 2 Department of Surgery, Division of Cardiothoracic Surgery, Medical College of Wisconsin, Milwaukee, Wisconsin, United States of America; University of Kansas Medical Center, United States of America

## Abstract

Hypoxia/Reoxygenation (H/R) cardiac injury is of great importance in understanding Myocardial Infarctions, which affect a major part of the working population causing debilitating side effects and often-premature mortality. H/R injury primarily consists of apoptotic and necrotic death of cardiomyocytes due to a compromise in the integrity of the mitochondrial membrane. Major factors associated in the deregulation of the membrane include fluctuating reactive oxygen species (ROS), deregulation of mitochondrial permeability transport pore (MPTP), uncontrolled calcium (Ca^2+^) fluxes, and abnormal caspase-3 activity. Erythropoietin (EPO) is strongly inferred to be cardioprotective and acts by inhibiting the above-mentioned processes. Surprisingly, the underlying mechanism of EPO's action and H/R injury is yet to be fully investigated and elucidated. This study examined whether EPO maintains Ca^2+^ homeostasis and the mitochondrial membrane potential (ΔΨ_m_) in cardiomyocytes when subjected to H/R injury and further explored the underlying mechanisms involved. H9C2 cells were exposed to different concentrations of EPO post-H/R, and 20 U/ml EPO was found to significantly increase cell viability by inhibiting the intracellular production of ROS and caspase-3 activity. The protective effect of EPO was abolished when H/R-induced H9C2 cells were treated with Wortmannin, an inhibitor of Akt, suggesting the mechanism of action through the activation Akt, a major survival pathway.

## Introduction

Acute myocardial Infarction (AMI) is a major cause of premature mortality in developed countries and is largely associated with Ischemia/Reperfusion (I/R) injury, which is the irreversible damage caused to myocytes during infarction [1]. AMI treatments, such as bypass surgery, are inefficient in addressing the symptoms of I/R injury, leading to complications. These complications primarily include apoptotic and necrotic cell death in myocytes due to an increase in mitochondrial reactive oxygen species (ROS) and unregulated calcium (Ca^2+^) fluxes [2]. These Ca^2+^ fluxes are also known to cause mitochondrial permeability transition pore (MPTP) to dysfunction causing an acute decrease in mitochondrial membrane potential (ΔΨm) thus further accelerating cell death [3], [4].

Erythropoietin (EPO) is a hematopoietic cytokine, and its receptor (EPOR) is shown to be present in tissues outside blood, including the heart. EPO, also possess a non-hematopoietic action, mediated through inhibition of apoptosis and appears to be essential for the tissue-protective effects of erythropoietin [5]. EPO, known for its protective role in hypoxic conditions, has shown protective properties against I/R injury by effectively reducing apoptotic renal cell death in in-vivo and in-vitro models [6]–[9]. EPO also showed a protective effect in neural cells by maintaining ΔΨm and intracellular Ca^2+^ concentration under pathological conditions [10]. It inhibits caspase-3, 8, 1 like activities and has been shown to protect against apoptosis and necrosis in in-vitro and in-vivo models of brain and spinal cord ischemic injury [11]–[13].

Studies have shown that in-vivo administration of recombinant human EPO reduces apoptosis and increases functional recovery after coronary artery occlusion/reperfusion [7], [14], [15]. EPO treatment prevented apoptosis of endothelial cells in-vitro through PI3K/Akt phosphorylation during Hypoxia [16]. It also activates the phosphorylation of STAT-5 and MAPK in these cells [16]. Recent work suggests that EPO application in microglia maintained the expression of Wnt1 thereby regulating the mitochondrial membrane potential, phosphorylation of BAD, inhibits caspase-1 and caspase-3 activation [17]. EPO has been shown to play an important role in cardio protection in rats, pigs and rabbits which are subjected to reperfusion injury by inhibiting apoptosis via activation of PI3K, Akt and Erk [15], [18]. The cardioprotection against necrotic cells and the rise in intracellular Ca^2+^ homeostasis, ROS, ΔΨm and the signaling by which this occurs is also not clear in cardiomyocytes. This study serves to investigate and elucidate the above-mentioned mechanisms of Hypoxia/Reperfusion (H/R) injury protection in H9C2 cells. We for the first time showed the protective effect of EPO in maintaining ΔΨ_m_ and intracellular Ca^2+^ homeostasis in live H9C2 cells, which were subjected to H/R to simulate the conditions of I/R. In summary, the EPO treatment attenuated apoptosis and necrosis through decrease in ROS, maintained ΔΨm and intracellular Ca^2+^ homeostasis via modulation of Akt pathway.

## Materials and Methods

### Characterization of H9C2 by Cardiac Specific Marker

H9C2, myoblast cell line derived from embryonic BD1X rat heart tissue was purchased from National Centre for Cell Science, Pune. Dulbecco's Modified Eagle's Medium (DMEM) with Fetal Bovine Serum (FBS) to a final concentration of 10% was used for this cell line. Cells were grown to about 70–80%confluency in a chamber slide and were fixed by using 3% paraformaldehyde and permeabilized in 0.1% of Triton-X 100 for 15 mins. Cells were incubated for 45 mins at 37°C with monoclonal α-sarcomeric actininin (A7811) (Sigma Aldrich) at a dilution of 1∶800 in PBS. Samples were washed and incubated with anti-mouse biotinylated secondary antibodies (Santa Cruz Biotechnology; 1∶200 dilutions) for 30 mins at 37°C. After washing with PBS, samples were incubated for 15 mins at 37°C with avidin-conjugated 1∶500 FITC. Samples were washed again with PBS, and the cover slip was placed over the chamber slide. Images were captured by confocal microscopy (Carl Zessis, ZEN 2010) using appropriate filters for visualization of Fluorescein isothiocyanate (FITC) (Excitation 490 nm and Emission 525 nm).

### Treatment of cells

H9C2 cells were cultured to 70–80% confluency and then serum starved in basal medium (DMEM + 0.1% Bovine Serum Albumin) for 24 hrs. H9C2 cells received no intervention (normoxic controls) or were exposed to H/R after pretreatment with two applications of EPO (10 U/ml, 15 U/ml and 20 U/ml). Treatment of EPO was accomplished 24 hrs before (first application) hypoxia. Second application during the induction of hypoxia and this hypoxic conditions were obtained by an incubation at 37°C in a small airtight chamber containing H9C2 cells with 94% N2, 5% CO_2_ remaining 1% O_2_ for 8 hrs in the presence of serum and glucose free DMEM medium[19]. Hypoxic medium were discarded and cell were reperfused with glucose containing DMEM + 10% FBS for 16 hrs. For some of the experiments, cells were treated with 1µM of Wortmannin before 30 mins of each application of EPO. Throughout the experiments control cells were maintained in DMEM+10% FBS.

### Cell viability assay

For determination of viability, cells were seeded in 96-well plates to 70–80% confluency. Followed the treatment procedure as described earlier, the medium was replaced with 100 µL of fresh phenol red free culture medium. 10 µL of the 12 mM MTT (3-(4, 5-dimethylthiazol-2-yl) -2, 5 diphenyltetrazolium bromide stock was added to each well. A negative control of 10 µL of the MTT stock solution was added to 100 µL of medium alone and incubated for 4 hrs at 37°C. After labeling the cells with MTT, medium was removed from the wells and 50 µL of DMSO were added and incubated at 37°C for 10 mins and read absorbance at 540 nm (Molecular Probes).The MTT assay involves the conversion of the water soluble MTT to an insoluble formazan. The amount of blue formazan dye generated from MTT was proportional to the number of live cells. The values of the reaction were obtained after subtraction of matched blanks and the ODs of the controls were taken as 100% for comparisons [19].

### Detection of Apoptosis by Confocal microscopy

Cells were grown to about 70–80% confluency and subjected to H/R or normoxia with or without pre-treatment with EPO as described earlier. The cells were then washed with PBS and collected by centrifugation and stained with a combination of Acridine Orange (Ao) (100 µg/ml, Excitation 502 nm and Emission 526 nm);Ethidium Bromide (EtBr) (100 µg/ml, Excitation 510 nm and Emission 595 nm) 1∶1 ratio for 10 mins and 10µl of the cell suspension was taken on slide and fluorescent images were scanned using a confocal laser scanning [20].

### Detection of Reactive oxygen species

#### Laser-scanning Confocal Microscopy

Cells were grown to 80% confluency in six-well plates and subjected to 8 hrs hypoxia and 15 mins reperfusion or normoxia with or without pre-treatment with EPO as described earlier. Cells were incubated with 100 µM of 1 ml 2′, 7′-Dichlorofluorescein Diacetate (DCF-DA, Molecular Probes) for 30 mins [21]. After incubation, cells were washed twice with PBS and imaged using confocal microscopy to detect ROS (Excitation 495 nm and Emission 520 nm).

#### Spectrofluorometry

To quantify intracellular ROS levels, Cells were cultured in 60 mm dishes to 70–80% confluency, and subjected to H/R or normoxia with or without pre-treatment with EPO as described earlier. Treated cells were incubated with 100 µM of 1 ml DCF-DA for 30 mins in a 5% CO2 and 37°C. Cells were washed twice with PBS and were lysed with 500µl 90% DMSO 10% PBS for 10 mins at room temperature in the dark [22]. Then 200µl of cell lysate was collected and the DCF fluorescence was immediately measured in a 96-well plate at 495 nm excitation and 520 nm emissions.

### Detection of Mitochondrial Membrane Potential

H9C2 cells pretreated with EPO were cultured on cover slips, and were exposed to 8 hrs hypoxia and 15 mins reperfusion as described earlier. After treatment cells were loaded with 5µg/ml of Rhodamine-123 and 100µM of DCF-DA for 30 mins in dark at room temperature [23]. Stained cells were washed thrice with PBS and cover slips were removed and inverted over the glass slide and images were captured under a confocal microscope. The laser power were adjusted to 2% to avoid bleaching, detector gain, and resolution of 512×512 were fixed in 40× oil immersion [24]. The same parameters were used for all the samples. Rhodamine-123 fluorescence was examined by illumination at 514 nm and detection at 570 nm and DCF fluorescence were measured as given above.

### Calcium live cell imaging

Cells were grown to 80% confluency in six-well plates and subjected to 8 hrs hypoxia and 15 mins reperfusion or normoxia with or without pre-treatment with EPO as described earlier. Cells with or without pretreatment of EPO were incubated with 4 µM of cell permeant Fluo-4 AM in dark before induction of hypoxia (Fluo-4 acetoxymethyl ester; Molecular Probes). After 8 hrs of hypoxia cells were visualized under confocal microscope and live cell images were taken in time series mode of about 0.2 secs for 15 mins [4]. Hypoxia medium were replaced by fresh DMEM + 10% FBS to reoxygenate those cells and live cell images were taken for 15 mins of reperfusion. Control cells were incubated with 4 µM of permeant Fluo-4-AM for 15 mins and live cell images were taken (excitation at 485 nm, emission at 520 nm).

### Immunofluorescence analysis

Cells were cultured in 4 well chamber slide to 70–80% confluency, and subjected to 8 hrs hypoxia and 30 mins reperfusion or normoxia with or without pre-treatment with EPO as described earlier. At the end of 30 mins reperfusion cells were washed with PBSfor three times and were fixed in 4% paraformaldehyde, permeabilized in methanol (−20°C), and incubated overnight at 4°C with primary monoclonal antibody for Phospho-Akt (Ser 473; Cell Signaling) at a dilution of 1∶500 in PBS. Samples were washed thrice with PBS and incubated for 30 mins at 37°C with anti-mouse biotinylated secondary antibodies (Santa Cruz Bio technology; 1∶500 dilutions). After washing cells were incubated for 15 mins at 37°C with avidin-conjugated FITC at a dilution of 1∶1000. Samples were washed again with PBS, and DAPI 1∶1000 dilution were added and incubated for 5 mins washed and mounted on microscopic cover slip [19]. Images were captured using confocal microscopy using appropriate filters for visualization of FITC and DAPI (described above).

### Western Blot analysis

H9C2 cells were cultured in 60 mm dishes to 80% confluency and were treated under normoxic conditions or 8 hrs hypoxia and 30 mins reperfusion with or without EPO and 1 µM Wortmannin before 30 mins of each application of EPO. Cells were kept on ice and washed thrice with ice cold PBS. Proteins were solubilized and extracted with 100 µl RIPA buffer (50 mM Tris pH 8.0, 150 mM NaCl, 0.5%SDS, 1% Nonidet P40, 0.5% sodium deoxycholate, 1 mM EDTA, 1X protease and phosphatase inhibitor cocktail (Cell Signaling technologies). The lysate were used to estimate protein content with the Bradford Assay Reagent. Equal amounts of protein (10–60 µg) from each sample were electrophoresed on a 10% SDS-polyacrylamide gel with running buffer and transferred to nitrocellulose membrane. The membranes were treated with primary antibody for p-AKT (1∶1000 dilutions, Cell Signaling Technologies) for overnight incubation at 4°C. They were again washed 3 times before incubating with matched secondary antibody (1∶5000) for 45 mins. The protein bands were developed with Alkaline Phosphatase substrate.

### Caspase-3 colorimetric assays

Cells were cultured in 60 mm dishes to 70–80% confluency, and subjected to H/R or normoxia with or without pre-treatment with EPO as described earlier. At the end of treatment the cells were collected by centrifugation, washed twice with ice-cold PBS and resuspended in lysis buffer as given in the caspase-3 colorimetric Assay kit (BF3100) R & D systems. The cell lysate was incubated on ice for 10 mins and centrifuged at 10,000-x g for 1 mins. The supernatant was transferred to a new tube and kept on ice. In 96-well plates, 50 µl (2 mg/ml) of cell lysate were added and 50 µl of 2X Reaction Buffer with 10 µl of fresh DTT stock per 1 ml of 2 X Reaction Buffer. Then 5µl of caspase-3 colorimetric substrate (DEVD-pNA) were added to each reaction well andincubated at 37°C for 1–2 hrs and measured the readings on a microplate reader at a wavelength of 405 nm.

### Statistical Analysis

Numerical data were presented as means ± SEM from three to five samples in all the experiments. The difference between groups was analyzed using ANOVA followed by TUKEYs test when permitted. Values for P< 0.001 were considered as statistically significant.

## Results

### Characterization of H9C2 cells by Cardiac Specific Marker

**Figure 1:**
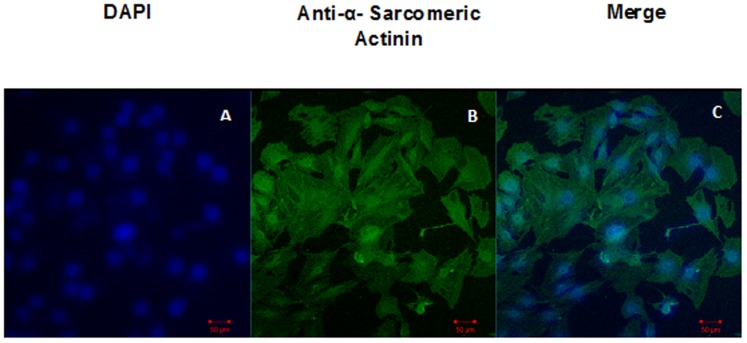
Characterization of H9C2 cells by Anti α – Sarcomeric actin. Panel A shows DAPI, Panel B shows α-sarcomeric actinin and Panel C shows merged image of DAPI and α-sarcomeric actinin.

The confocalmicroscope images showed positive stain for Anti α-sarcomeric actin antibody (Figure 1) the cardiac specific marker.

### EPO treatment increases cell viability

**Figure 2:**
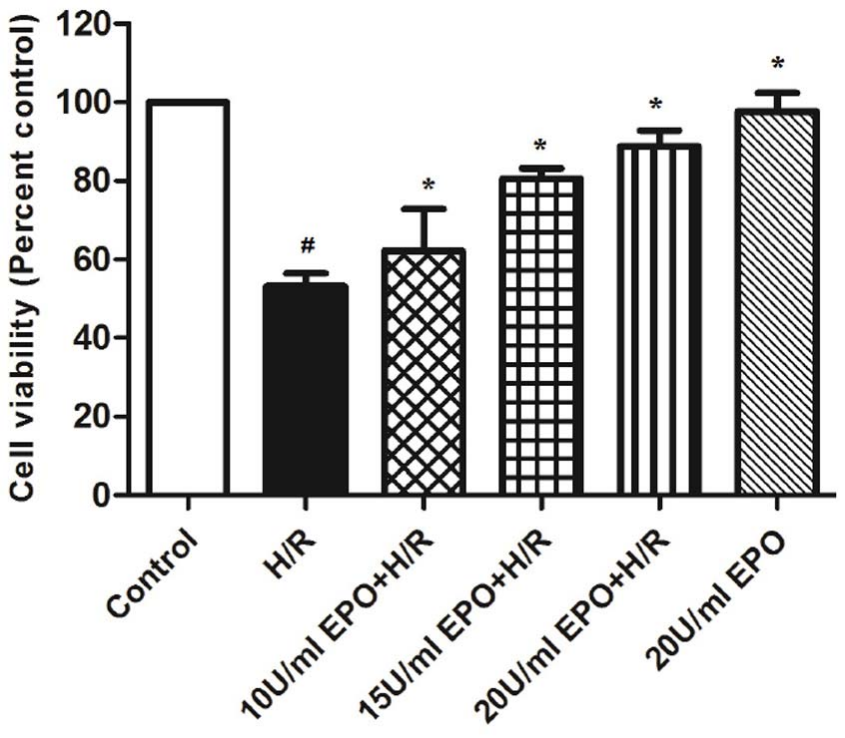
Pre-treatment of EPO increases cell viability in H/R induced H9C2 cells. The effect of EPO on cell viability was determined using MTT assay. H9C2 cells were subjected to H/R with or without pre-treatment with (10 U/ml, 15 U/ml and 20 U/ml) EPO for 24 hrs. 20 U/ml EPO significantly increases cell viability after H/R. Data are presented as means ± SEM of the ratios from five independent experiments. ^$^denotes p<0.05, ^&^ denotes p<0.01, * denotes p<0.001 for analyses compared to H/R.

MTT assays were performed to evaluate cell viability, and the control was used as a maximum reference (100%) to calculate the effect of EPO treatments on post-H/R cell viability. The H/R-induced cells were pretreated with increasing concentrations of EPO (10, 15 and 20 U/ml) and showed a maximum of 88% cell viability with 20 U/ml as opposed to the 53% viability seen in cells that were solely subjected to H/R treatment (Figure 2). Based on these data, we further used 20 U/ml of EPO concentrations in all of our experiments.

### EPO treatment reduces H/R-induced cell death

**Figure 3:**
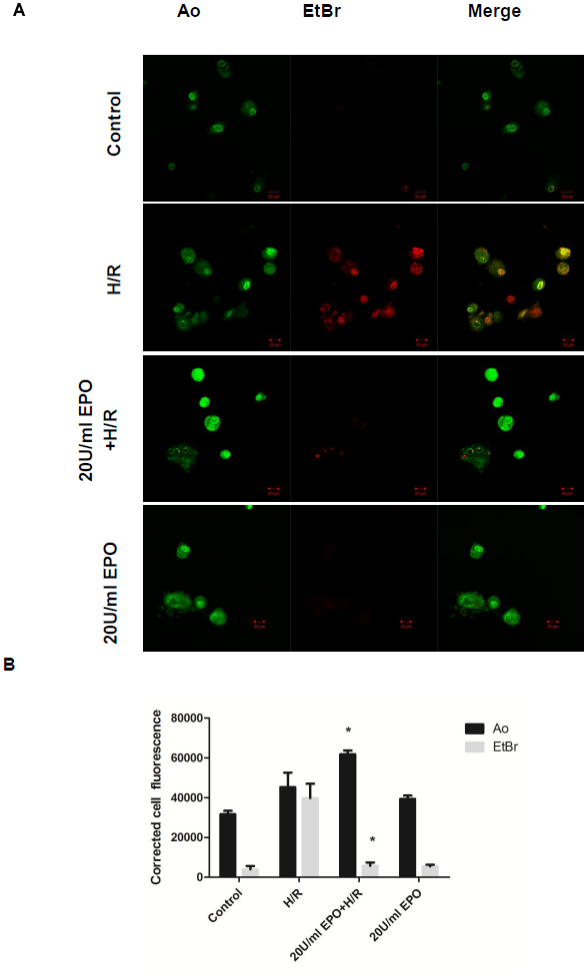
Pre-treatment of EPO in H/R induced H9C2 cells had uniformly green nuclei with intact plasma and nuclear membranes. The effect of EPO on apoptosis and necrosis was determined using Acridine/Orange (Ao/EtBr) double staining assay. H9C2 cells were subjected to H/R with or without pre-treatment with 20 U/ml EPO for 24 hrs. (A) EPO stabilizes plasma membrane and nuclear membrane showed byuniform green nuclei. In H/R induced cells, early apoptotic cells were indicated by intact membrane but nuclei was condensed and green. Late-stage apoptotic cells were indicated by bright orange-stained nuclei. Necrotic cells were indicated by red stained nuclei. (B) Represents the quantification of the above image. Data are presented as means ± SEM of the ratios from three independent ratios *p<0.001 for analyses compared to H/R.

Double staining with Ao and EtBr allowed to differentiate between live, apoptotic and necrotic cells [25]. Both viable and dead cells take up Ao whereas EtBr was excluded from living cells. Late apoptotic or necrotic cells have ruptured nuclear membrane that allows the entrance of EtBr to intercalate with DNA [26], [27]. In our study, control cells were seen as bright green colored nuclei with intact and uniform cell membranes. Cells subjected to H/R showed some early apoptotic, late apoptotic and necrotic nuclei. Early apoptotic cells have a green condensed, shrunken or fragmented nucleus, whereas late apoptotic showed uniform orange nuclei and necrotic cells showed red nuclei. Accordingly cells pretreated with 20 U/ml of EPO before H/R showed green nuclei confirmed the cardiomyocyte protection form H/R injury (Figure 3).

### EPO treatment reduces H/R-induced ROS production

**Figure 4:**
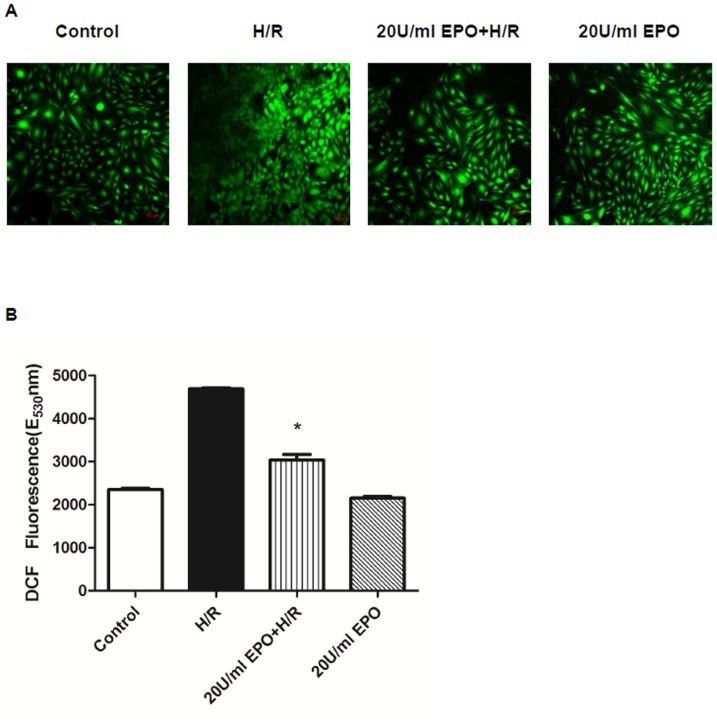
Pre-treatment of EPO decreases ROS generation in H/R induced H9C2 cells. The effect of EPO on ROS generation was determined using DCF-DA assay in confocal Microscopy (A), spectrofluorometry (B). H9C2 cells were subjected to H/R with or without pre-treatment with 20 U/ml EPO for 24 hrs, H/R induced cells showed increase in green fluorescence (A), more fluorescence intensity (B), when compared to control and 20 U/ml EPO treated. Thus EPO decrease ROS generation after H/R. Data are presented as means ± SEM of the ratios from three independent experiments * denotes p<0.001 for analyses compared to H/R.

2′, 7′-dichlorofluorescin Diacetate (DCFH-DA) is a nonfluorescent substrate that crosses the cell membranes upon deacetylation by esterases and oxidation by ROS in the cytoplasm. As soon as DCFH-DA is converted into DCF in the cytoplasm, green fluorescence is produced, which is directly proportional to the intracellular ROS production [28]–[30]. The intracellular production of ROS was detected using confocal microscopy and spectrofluorometry. The control cells, cells treated with 20 U/ml EPO, and cells pretreated with 20 U/ml EPO before H/R, showed lesser green fluorescence compared to cells subjected to H/R alone. Fluorescence intensity was quantified using confocal and spectrofluorometric analysis (Figure 4A, B). The results showed that EPO pretreatment decreases ROS production, and EPO act as an anti-oxidant to regulate ROS production post- H/R.

### EPO treatment stabilizes ΔΨ_m_ in H/R-induced cells

**Figure 5:**
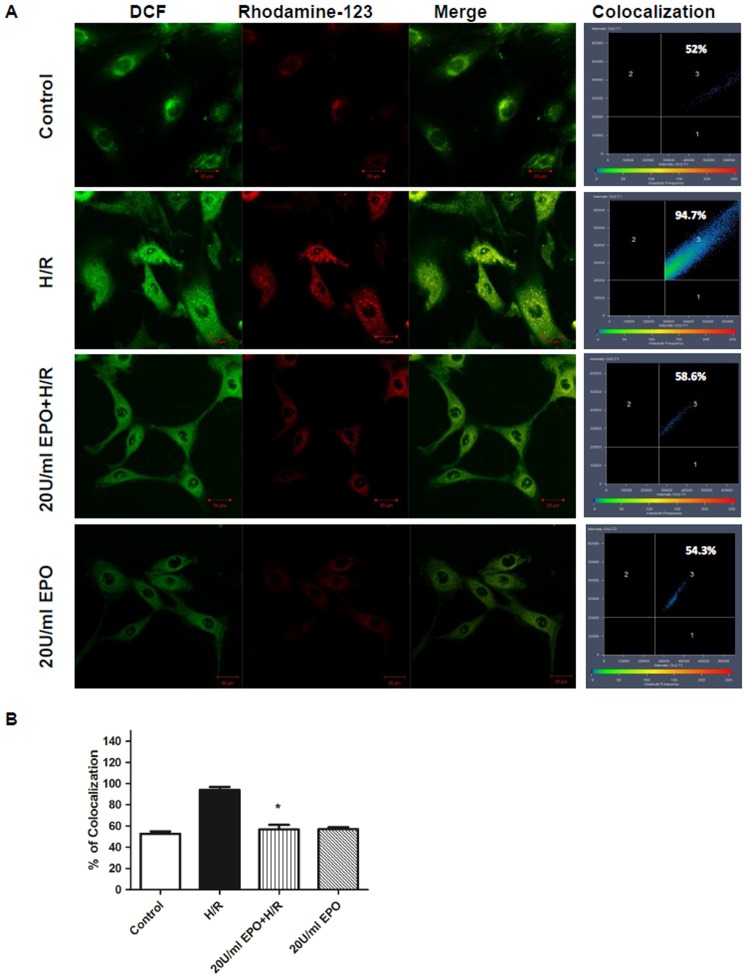
Pre-treatment of EPO maintains mitochondrial membrane potential in H/R induced H9C2 cells. The effect of EPO on Membrane potential was determined by colocalization studies using Rhodamine-123 and DCFH-DA. H9C2 cells were subjected to H/R with or without pre-treatment with 20 U/ml EPO for 24 hrs. H9C2 cells pretreated with EPO and control cells showed Rhodamine-123 fluorescence only in the perinuclear region about 58% and 52% colocalzation respectively whereas H9C2 cells subjected to H/R showed Rhodamine-123 fluorescence both in perinuclear region and cytosol about 94.7% co localization in Figure 5 A. Data are presented as means ± SEM of the ratios from three independent experiments * denotes p<0.001 for analyses compared to H/R.

In the control and EPO treated H9C2 cells, red fluorescence was emitted by Rhodamine-123 and it appeared exclusively in the perinuclear region of the cells. These are the regions where mitochondria are localized [31]. Green fluorescence appeared in both in the cytosol and the mitochondria as DCFH-DA is able to diffuse across both cell and mitochondrial membranes, as previously mentioned. In H/R induced cells, MPTP opens and there is an influx of Ca^2+^ ions into the mitochondria. The accumulation ruptures the outer mitochondrial membrane and Rhodamine-123 leaks out from the mitochondria into the cytosol. Rhodamine-123 and DCF fluorescence were observed to co-localize exclusively in the perinuclear regions of the control and EPO treated cells. While in H/R induced cellsRhodamine-123 co-localized in the perinuclear and the cytoplasmic regions (Figure 5A).

The scatter plot illustrating the degree of co-localization between the two dyes showed a significant increase of about 94.7% in H/R induced cells. Whereas, the control and EPO pretreated cells showed 52% and 58%co-localization, and it is significantly lower when compared to H/R induced cellsas seen in the scatter plot (Figure 5B). These results clearly indicate that EPO stabilizes ΔΨ_m_ in H/R-induced cells.

### EPO treatment maintains mitochondrial membrane integrity and intracellular Ca^2+^ homeostasis

**Figure 6:**
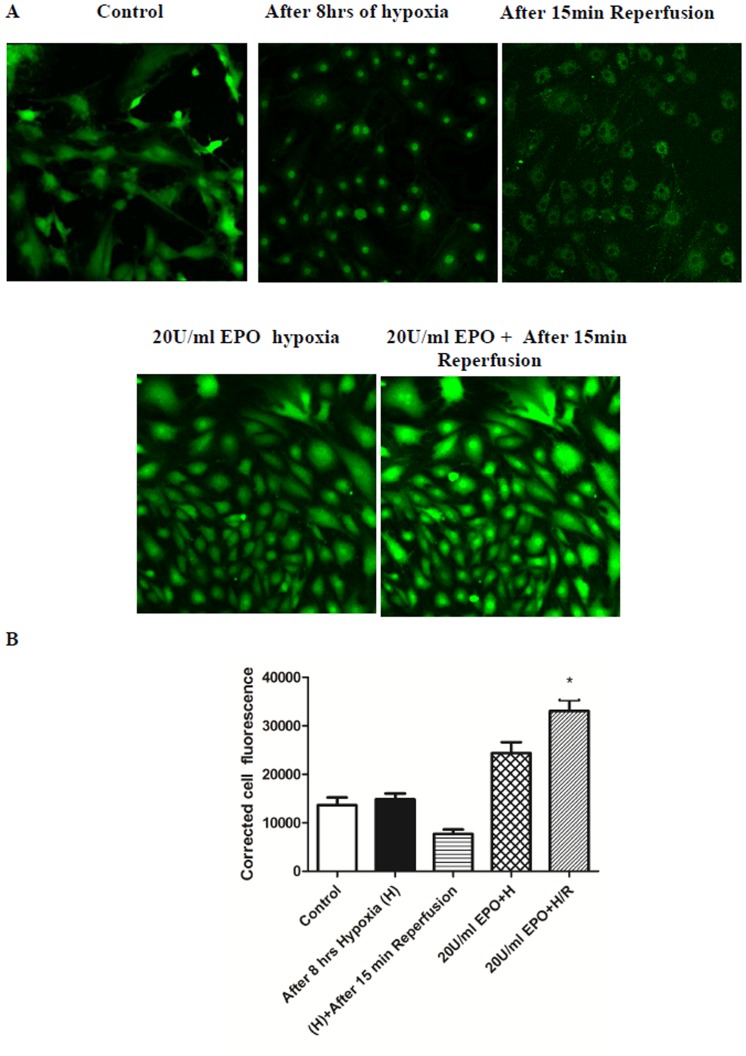
Pre-treatment of EPO maintains intracellular calcium and prevents MPTP opening in H/R induced H9C2 cells. The effect of EPO on intracellular calcium and MPTP opening was determined using time–lapse imaging in confocal microscope. (A) H9C2 cells were subjected to 8 hrs hypoxia and 15 mins reperfusion with or without pre-treatment with 20 U/ml EPO for 24 hrs and stained with Fluo-4 AM and performed live cell imaging. H9C2 cells pretreated with EPO accumulate Ca^2+^ inside the mitochondrial membrane even after reperfusion and showed increase Fluo-4 AM fluorescence. H9C2 cells subjected to H/R showed release of Ca^2+^ outside the mitochondria due to overload of Ca^2+^ during reperfusion rupture of mitochondrial membrane occurs. (B) Represents the quantification of Fluo-4 AM fluorescence. Data are presented as means ± SEM of the ratios from three independent experiments * denotes p<0.001 for analyses compared to H/R.

Normal cells maintain ΔΨ_m_ by accumulating Ca^2+^ both in the cytosol and the nucleus, as visualized by Fluo-4 AM in live cell imaging (Video S1). In cells with 8 hrs of hypoxia exposure, Ca^2+^ accumulates more in the mitochondria and the nucleus compared to the cytosol. At the start of reperfusion MPTP open and the mitochondrial membrane ruptures due to a Ca^2+^influx intothe mitochondria and the nucleus (Video S2). In contrast, cells pretreated with EPO maintained mitochondrial membrane integrity and intracellular Ca^2+^ homeostasis (Figure 6, Video S3)

### EPO induces phosphorylation of Akt and reduces caspase-3 activity in H/R-induced cells

**Figure 7:**
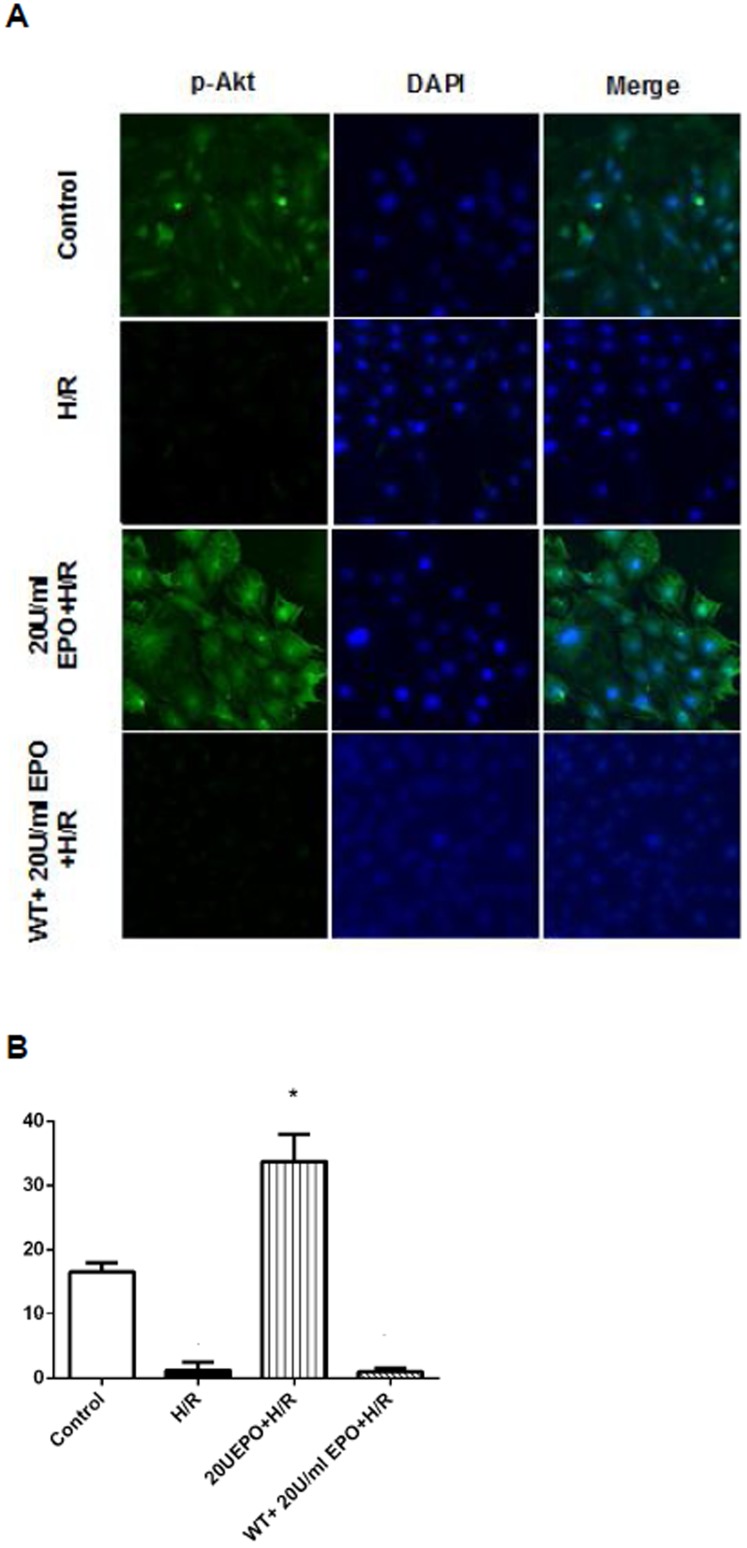
Pre-treatment of EPO increases intracellular p-Akt in H/R induced H9C2 cells. The effect of EPO on p-Akt was determined. H9C2 cells (A) were subjected to 8 hrs hypoxia and 30 mins of reperfusion with or without pre-treatment with 20 U/ml EPO and 1 µM Wortmannin before 30 mins for 24 hrs and stained with p-AKT antibody. EPO increase p-Akt. (B) Represents the quantification green florescence, which indicates the increase in levels of p-Akt. Data are presented as means ± SEM of the ratios from three independent experiments * denotes p<0.001 for analyses compared to H/R.

**Figure 8:**
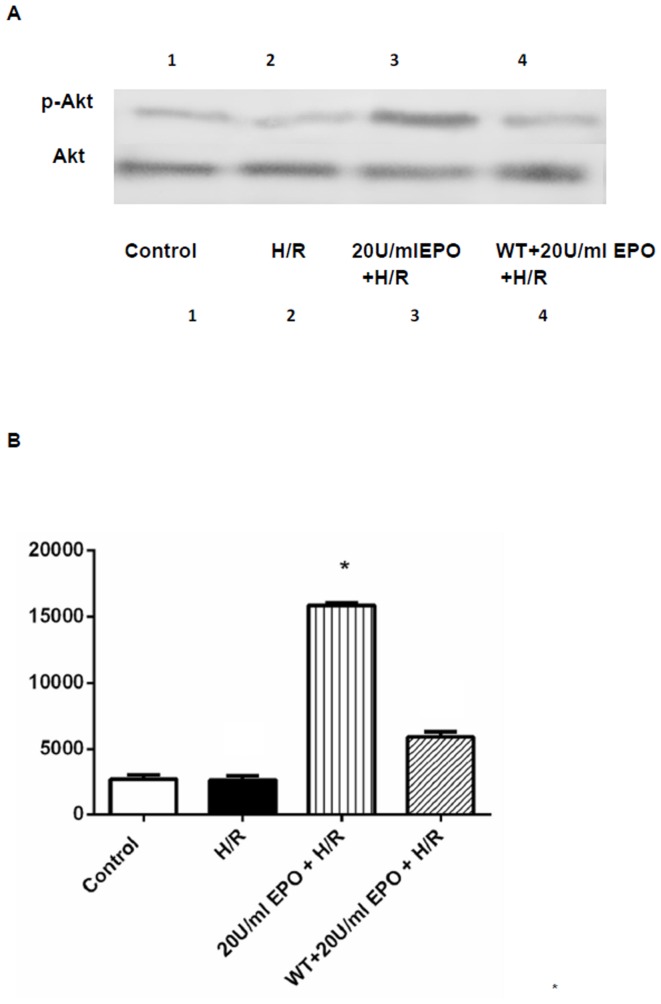
Western blot analysis demonstrating the effect of EPO on AKT. The effect of EPO on p-Akt was determined using Western blot. H9C2 cells were ^i^subjected to H/R with or without pre-treatment with 20 U/ml EPO for 24 hrs and incubated with 1 µM of Wortmannin 30 mins before EPO treatment. (A) Samples treated with EPO showed increase in phosphorylation of Akt (p-Akt). Akt remain unaltered and demonstrate equal protein loading in all lanes. (B) Represent the quantization of western blot, which indicates the increase in p-Akt levels Data are presented as means ± SEM of the ratios from three independent experiments * denotes p<0.001 for analyses compared to H/R.

Akt is involved in cellular survival pathway by inhibiting apoptotic processes. On the other hand, caspase-3 is playing an important role in activating the final step of the proapoptotic-signaling pathway in many cell lines. To examine the role of Akt and caspase-3 in EPO treatment mediated protection in H/R-induced cell injury; we performed Western and immunofluorescence analysis for p-Akt and spectrophotometric analysis for caspase-3. The immunofluorescence analysis showed an increase in levels of p-AKT (green) as compared to the H/R-induced cells and normoxic controls (Figure 7A, B). The increase in p-Akt was further confirmed by Western analysis from H9C2 cells pretreated with 20 U/ml EPO (Figure 8).

**Figure 9:**
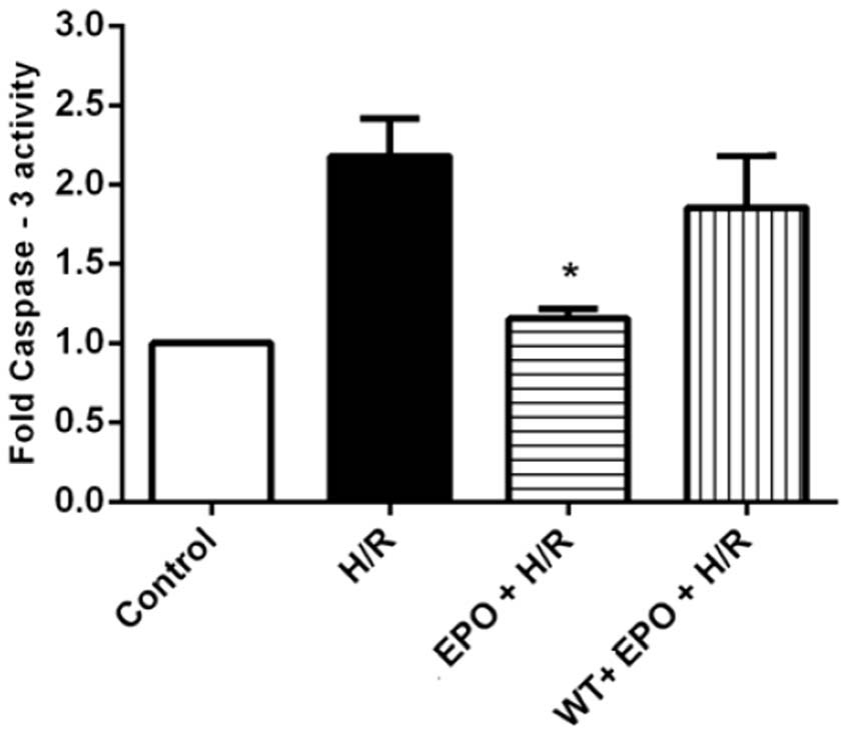
Pre-treatment of EPO decreases caspase-3 activity in H/R induced H9C2 cells. The effect of EPO on caspase-3 activity was determined using caspase-3 colorimetric assay. H9C2 cells were subjected to H/R with or without pre-treatment with 20 U/ml EPO for 24 hrs and incubated with 1 µM of Wortmannin 30 mins before EPO treatment. EPO decrease caspase-3 activity after H/R. H9C2 cells pretreated with wortmannin showed increase in caspase-3 activity. Data are presented as means ± SEM of the ratios from three independent experiments * denotes p<0.001 for analyses compared to H/R.

Caspases are cysteine proteases, which play an essential role in apoptosis, necrosis and inflammation. To examine whether EPO exerts anti-apoptotic actions on H/R-induced H9C2 cells, we used caspase-3 specific peptide reporter molecule, p-nitro aniline (pNA), to cell's lysate and quantified by spectrophotometer at 405 nm. The color intensity is directly proportional to the level of caspase-3 activity in the cell lysate. Caspase-3 activity increased two fold in H9C2 cells after exposure to H/R, whereas, there was a significant decrease in caspase-3 activity in cells that were pretreated with 20 U/ml of EPO (Figure 9). To further validate the significance of p-Akt and caspase-3 in EPO-treatment mediated protection, we next inhibited Akt using specific inhibitor Wortmannin. We observed that EPO-treatment induce phosphorylation of Akt was inhibited by the treatment Wortmannin (Figure 7, 8). Furthermore, reduced caspase-3 activity by H/R-induced EPO treatment effect was blunted on treatment with Wortmannin. Together, these data indicate that protective effect of EPO-treatment in H/R-induced cell injury is mediated through the suppression of pro-apoptotic caspase-3 activity and activation of survival p-Akt signaling pathway (Figure 7-9).

## Discussion

Our results demonstrate for the first time that the protective effect of EPO in maintaining ΔΨ_m_ and intracellular Ca^2+^ homeostasis in live H9C2 cells, which were subjected to H/R to simulate theconditions of I/R.In our study,the20 U/ml of EPO showed 80% protection of H9C2 cells induced with H/R as compared to 50% protection when treated with 0.4 U/ml or 10 U/ml of EPO [15]. The results of this study also strongly support the hypothesis that EPO pretreatment protects cardiomyocytes from H/R induced apoptosis and necrosis by stabilization of ΔΨ_m_, ROS and Ca^2+^ overload via modulation of Akt and caspase-3 activity.

Mitochondria plays a key role in ROS generation during both ischemia and reperfusion in the heart [3]. The main factorsduring the propagation and execution phases of apoptosis and necrotic cell death are increased ROS and Ca^2+^ overload in the mitochondria due to decreasing ΔΨ_m_ [32]. It is quite evident that apoptosis is characterized by membrane blebbing, chromatin aggregation, DNA condensation, whereas, necrosis is characterized by cell swelling, cell lysis, disruption of membrane integrity & organelle damage [33]. The activation of caspase-3 protease primarily acts downstream in mitochondrial pathways by triggering apoptotic and necrotic cell death. ROS may act as an initiatorin this process by cleaving poly ADP-Ribose polymerase (PARP), the 70 KD protein of the U1-ribonucleoprotein and a subunit of the DNA dependent protein kinase [34], [35]. The ROS activated caspase-3 cleaves PARP at the amino acid motif site DEVD triggering apoptosis [33], [36], [37]. Inhibition of these processes was in turn inferred to reduce cell death, and EPO was successfully used in this study in demonstrating significant reductions in the levels of ROS and caspase-3 activation in H9C2 cells upon exposure to H/R.

Mitochondrial dysfunction has been suggested to play a central role in apoptotic and necrotic pathway. Thus the opening of MPTP during apoptosis and necrosis has been demonstrated to induce depolarization of transmembrane potential i.e., decrease in ΔΨ_m_ [38]. Rhodamine-123 is a commonly used indicator of ΔΨ_m_, and it distributes passively between cytosol and mitochondria - depending on the membrane potential [23]. Due to the Ca^2+^overload inside the mitochondria, the rupture of outer mitochondrial membrane and subsequent leakage of Rhodamine-123 dye from mitochondria to cytosol occurs in H/R induced cells. Unlike in H/R induced cells, Rhodamine-123 accumulated only in mitochondria of control cells and cells pretreated with 20 U/ml of EPO. Accordingly, cells, which were subjected to H/R, showed a decrease in ΔΨ_m_ due to the accumulation of Ca^2+^ inside the mitochondria, and the subsequent rupture of outer mitochondrial membrane. In contrast, the pre-treated cells showed intact mitochondrial membranes illustrating the protective effect against H/R. Further, the mechanistic evidence of EPO protection in live cell imaging showing the MPTP opening in H/R induced cells and the maintenance of intracellular Ca^+^ homeostasis in EPO pretreated cells after H/R has been clearly illustrated in this study. To reiterate, 20 U/ml EPO pretreatments maintains ΔΨ_m_ and intracellular Ca^2+^ homeostasis during H/R injury.

EPO anti-hypoxic trait is not limited to H9C2 cells and previous investigations demonstrates EPO's anti-apoptotic and anti-necrotic actions in other systems, includingthe brain,kidneys, and the liver, in which EPO acts against the apoptosis and necrosis caused by I/R [39], [40]. EPO (1000 IU/kg/day subcutaneously), (300 IU/kg intravenously) pre-treatment was able to attenuate the renal dysfunction and injury associated with I/R [41], [42].Administration of EPO in (1,000 IU/kg/day) rat model of type II collagen-induced arthritis tissue injury resulted in decreased proinflammatory cytokines in the circulation [43]. Additionally, during chronic hypoxia EPO (400 IU/kg) exerts significant beneficial effects when administered 18 hours post-cecal ligation and puncture (CLP) [14]. Short-term low-dose' EPO improved cardiac function and infarct size without any clinical adverse effects, but it did not prevent neointimal hyperplasia in percutaneous coronary intervention treated acute myocardial infarction patients [44]. Evidence suggests, EPO treatments protected adult rat cardiomyocytes in-vitro and in-vivo in a rat model of myocardial infarction [14]. EPO also prevents motor neuron apoptosis and neurological disability in spinal cord injury [13]. The neuroprotective effect of EPO is conferred by attenuating the production of ROS and reducing the basilar artery vasoconstriction [15], [45]–[50] on neural vascular endothelium [51].

EPO pretreatments showed a protective effect by decreasingcaspase-3 activity, and the mechanism of inhibition is inferred to be through the modulation of pro-survival signaling pathway Akt. Evidence of this mechanism was illustrated by blocking the Akt pathway by Wortmannin inhibitor and an increase in caspase-3 activity was observed compared to the control.Accordingly, protectionfrom apoptosis and necrosiswas abolished in cells solely treated with Wortmannin, thus confirming that the effect is primarily due to phosphorylation of Akt, the major survival pathway.

In conclusion, this study illustrates a novel finding thatshows the protective effect of EPO against apoptosis and necrosis in H9C2 cells subjected to H/R injury. It was found that the treatment with EPO protects cardiomyocytes by phosphorylation of Akt. This treatment also attenuates both the increase in activity of caspase-3, intracellular ROS induced by H/R and stabilizes ΔΨ_m_ and intracellular Ca^2+^ homeostasis. Together these findings support mechanistic evidence for the protective effect of EPO in cardiomyocytes to prevent H/R-induced cell death and possibly create new avenues for effective cardioprotective therapeutics.

## Supporting Information

Video S1 **Control H9C2 cells were incubated with 4 µM of cell permeant Fluo-4 AM in dark for 30 mins and kept under confocal microscope and images were taken in time series mode of about 0.2 secs for 15 mins.** Control cells maintain ΔΨ_m_ by accumulating Ca^2+^ both in the cytosol and the mitochondria.
(MP4)

Video S2 **After 8 hrs of hypoxia cells were visualized under confocal microscope and live cell images were taken in time series mode of about 0.2 secs for 15 min.** Hypoxia medium were replaced by fresh DMEM + 10% FBS to reoxygenate those cells and live cell images were taken for 15 mins of reperfusion. After 8 hrs of hypoxia exposure, Ca^2+^ accumulates more in the mitochondria and the nucleus compared to the cytosol. At the start of reperfusion MPTP open and the mitochondrial membrane ruptures due to a Ca^2+^influx into the mitochondria.
(MP4)

Video S3 **H9C2 cells pretreated with EPO before induction of H/R were visualized after 8hr of hypoxia and reperfused while taking the images in time series mode of about 0.2 secs for 15 mins.** In contrast, cells pretreated with EPO maintained mitochondrial membrane integrity and intracellular Ca^2+^ homeostasis.
(MP4)
