# Correction: Erythropoietin Protects Cardiomyocytes from Cell Death during Hypoxia/Reperfusion Injury through Activation of Survival Signaling Pathways

**DOI:** 10.1371/journal.pone.0115268

**Published:** 2014-12-04

**Authors:** The PLOS ONE Staff

In Figure 2, the symbols above the 3^rd^ and 4^th^ bars are incorrect. Please see the corrected Figure 2 here.

**Figure 2:**
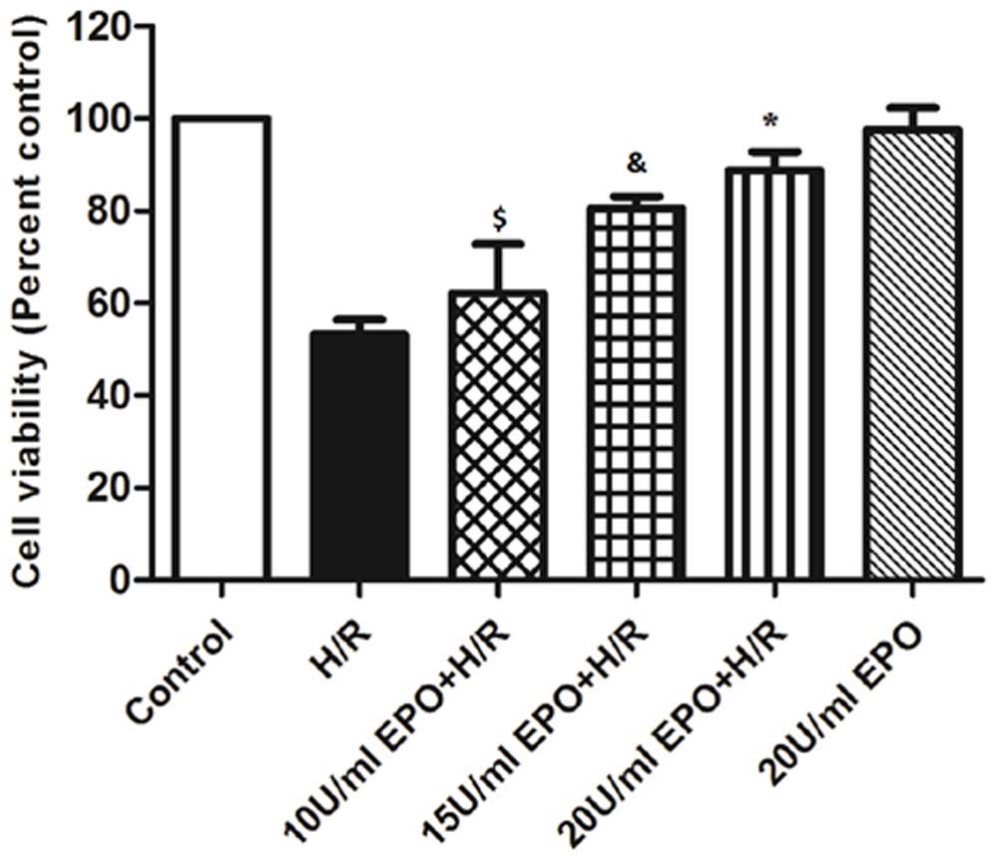
Pre-treatment of EPO increases cell viability in H/R induced H9C2 cells. The effect of EPO on cell viability was determined using MTT assay. H9C2 cells were subjected to H/R with or without pre-treatment with (10 U/ml, 15 U/ml and 20 U/ml) EPO for 24 hrs. 20 U/ml EPO significantly increases cell viability after H/R. Data are presented as means ± SEM of the ratios from five independent experiments. ^$^denotes p<0.05, ^&^ denotes p<0.01, * denotes p<0.001 for analyses compared to H/R.
